# Application value of tumor necrosis factor inhibitors in in vitro fertilization-embryo transfer in infertile women with polycystic ovary syndrome

**DOI:** 10.1186/s12884-023-05546-0

**Published:** 2023-04-13

**Authors:** Jun-xia Liang, Yu Zhang, Chun-hui Xiao, Shan Cao, Ying Tian, Na-na Wang, Chong Liu

**Affiliations:** 1 Reproductive Department, Hebei Institute of Reproductive Health Science and Technology, Shijiazhuang, 050071 Hebei China; 2 Obstetrics Department, The Fourth Hospital of Shijiazhuang, Shijiazhuang, 050033 Hebei China

**Keywords:** Tumor necrosis factor (TNF) inhibitors, Polycystic ovary syndrome (PCOS), In vitro fertilization-embryo transfer (IVF-ET), Application value, Infertility

## Abstract

**Background:**

Clinical value of tumor necrosis factor (TNF) inhibitors in in vitro fertilization-embryo transfer (IVF-ET) in infertile women with polycystic ovary syndrome (PCOS) was investigated in this study.

**Methods:**

A retrospective analysis was performed on the clinical data of 100 PCOS patients who received IVF-ET for the first time at Hebei Institute of reproductive health science and technology from January 2010 to June 2020. The patients were divided into Inhibitor group and Control group according to whether they were treated with or without TNF inhibitors. Next, the two groups were subject to comparison in terms of the days of gonadotropin (Gn) use, total dosage of Gn, trigger time, hormone level and endometrial condition on the day of human chorionic gonadotropin (HCG) injection, the effects of two different regimens on controlled ovarian hyperstimulation (COH) and pregnancy outcomes.

**Results:**

There were no significant differences in baseline characteristics between the two groups, including age, duration of infertility, body mass index (BMI), ovarian volume, antral follicle count, and basal hormone levels. Compared with the Control group, the days of Gn use and trigger time of patients in the Inhibitor group were significantly shortened, and the total Gn dosage was notably reduced. In terms of sex hormone levels on the HCG injection, the Inhibitor group displayed much lower serum estradiol levels while higher serum luteinizing hormone and progesterone (P) levels than the Control group. Notably, the high-quality embryo rate was also significantly increased with the use of TNF inhibitors. However, significant differences were not observed in endometrial thickness (on the day of HCG injection), proportion of endometrial A, B and C morphology (on the day of HCG injection), cycle cancellation rate, number of oocytes retrieved, fertilization rate, and cleavage rate between the two groups. Importantly, the clinical pregnancy rate in the Inhibitor group was significantly higher than that in the Control group, but there was no significant difference in the biochemical pregnancy rate, early abortion rate, multiple birth rate, ectopic pregnancy rate and number of live births between the two groups.

**Conclusion:**

Collectively, after application of TNF-α inhibitor regimen, superior overall treatment effect can be observed in infertile PCOS patients receiving IVF-ET. Therefore, TNF inhibitors have certain application value in IVF-ET in infertile women with PCOS.

## Background

Polycystic ovary syndrome (PCOS), characterized by complex causes and diverse clinical manifestations, is a gynecological endocrine disorder occurring in women of childbearing age and a major contributor to female infertility [1]. An epidemiological survey has reported the incidence of infertility (10% – 15%) [2] and PCOS (5% – 10%) in China; and notably, infertility caused by PCOS accounts for about 50% of total infertility [3]. PCOS-induced infertility brings a great economic burden and pressure on health resources in China. Fortunately, with the booming development of assisted reproductive technology, in vitro fertilization-embryo transfer (IVF-ET) has become an effective therapy for PCOS-induced infertility [4]. Controlled ovarian hyperstimulation (COH) is a key link in IVF-ET. Some studies have claimed that, despite 40% – 50% of implantation rate of IVF, the successful pregnancy rate is only 30% – 40% [4]. The reason for this phenomenon is poor embryo quality due to the difficulty in COH [4]. Therefore, finding a medication regimen characterized by short COH time, low gonadotropin (Gn) dosage, high-quality embryo rate, high pregnancy rate, and low abortion rate is of great significance for infertile PCOS patients.

Subclinical inflammation is an important factor in endocrine, metabolic, and reproductive disorders in PCOS. Several studies have observed increased expression of multiple inflammatory cytokines in PCOS patients, and among these cytokines, TNF-α has received particular attention [5–7]. TNF-α is a vital pro-inflammatory cytokine produced by macrophages of adipose tissue. With the increase in visceral adipose tissue, adipose tissues serve as an endocrine organ to promote the production of adipokines and the secretion of TNF-α, thereby participating in the development and progression of various diseases [8]. Studies have stated that TNF-α concentrations in serum and follicular fluid are increased in PCOS patients; TNF-α in serum can affect the occurrence of PCOS via different pathways, and inflammatory cytokines in follicular fluid may be closely related to follicular development and ovulation [9]. Through inhibiting the release of inflammatory factors, TNF-α inhibitors can delay the progression of endometriosis, improve joint inflammation and joint function, and delay the radiological progression of joints [10–12]. Due to the above findings, the possible role of TNF-α inhibitors in PCOS patients has been speculated. However, there are no relevant reports on whether TNF-α inhibitors can improve the clinical outcome of IVF-ET in PCOS patients. Therefore, we mainly compared the effects of two regimens (with or without TNF-α inhibitor) on ovulation induction outcome, sex hormone levels and pregnancy outcomes of PCOS patients who underwent IVF-ET in this study. Through a series of comparisons, this research further revealed the application value of TNF-α inhibitors.

## Materials and methods

### General information

**Fig. 1:**
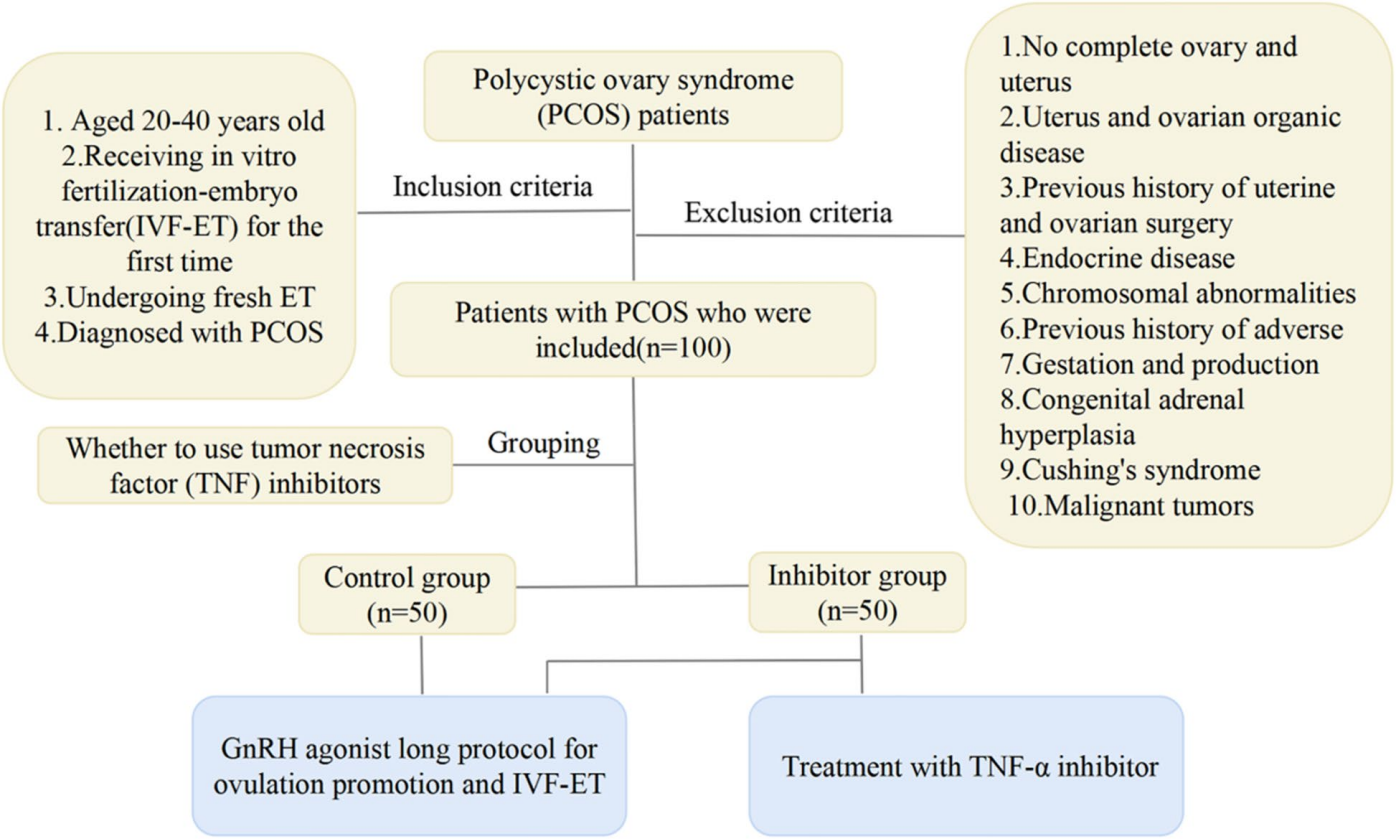
The flow chart of study

PCOS patients who received IVF-ET for the first time at Hebei Institute of reproductive health science and technology from January 2010 to June 2020 were selected. A total of 100 patients were finally included in this study after screening based on the inclusion and exclusion criteria. According to the application of TNF inhibitors, patients were divided into Inhibitor group (with TNF-α inhibitor, *n* = 50) and Control group (without TNF-α inhibitor, *n* = 50). All patients received a long protocol of Gn releasing hormone (GnRH) agonist for ovulation induction. This study was approved by the Ethics Committee of Hebei Institute of reproductive health science and technology. Figure 1 illustrates the study flow.

Inclusion criteria [13] of patients consisted of (1) aged 20–40 years old; (2) receiving IVF-ET for the first time and undergoing fresh embryo transfer; (3) diagnosed with PCOS according to Rotterdam 2003 criteria recommended by ESHRE/ASRM [14], i.e., the diagnosis met 2 items of the following 3 items: ① oligoovulation and (or) anovulation; ② clinical manifestations and (or) biochemical indicators of hyperandrogenism; ③ polycystic ovarian changes: B ultrasound showed at least one ovarian antral follicle (diameter 2–9 mm) count ≥ 12, and (or) ovarian volume > 10 ml. As for exclusion criteria, patients were excluded if they (1) had no complete ovary and uterus, or combined with uterus and ovarian organic disease, or had a previous history of uterine and ovarian surgery; (2) combined with hyperprolactinemia, endocrine disease; (3) had chromosomal abnormalities, previous history of adverse gestation and production, such as embryo arrest, hydatidiform mole, habitual abortion, fetal malformations, intrauterine fetal death; (4) combined with congenital adrenal hyperplasia, Cushing's syndrome, malignant tumors.

### Treatment

#### TNF-α inhibitor for treatment

Days 1–4 of the menstrual cycle prior to ovulation induction treatment, patients in the Inhibitor group were treated with TNF inhibitors until final oocyte maturation was triggered. Specifically, Recombinant Human Tumor Necrosis Factor-α Receptor: IgG Fc Fusion Protein for Injection (YISAIPU; 202,205,027, Sunshine Guojian Pharmaceutical (Shanghai) CO., Ltd., Shanghai, China) was subcutaneously injected into patients under the conditions of sterilization and water dissolution, 25 mg for each injection, administration twice each week, for an interval of 3–4 d.

#### Protocol for ovulation induction

Long protocol of GnRH agonist was adopted for ovulation induction of all patients. Before treatment, GnRHa short-acting triptorelin (Diphereline; U10084A, Ferring Pharmaceuticals (China) Co., LTD, Shanghai, China) was injected for the down-regulation of pituitary from mid-luteal phase of the menstrual cycle. After 14 d of injection, endometrial thickness and serum estradiol (E2), follicle-stimulating hormone (FSH) and luteinizing hormone (LH) levels were measured. Upon successful down-regulation, Gn (Gonal-f; S20160040, Merck, Germany) was injected 150–225 IU/d subcutaneously, and then the dosage of Gn was adjusted according to the follicular development monitored by B ultrasound.

#### In vitro fertilization-embryo transfer

All patients underwent IVF-ET. HCG 10000U trigger was administered at night when one dominant follicle ≥ 18 mm in diameter or two follicles ≥ 16 mm in diameter could be observed. After 36–38 h, puncture of oocyte retrieval was performed under the guidance of transvaginal ultrasound, followed by IVF. Next, the prokaryotic expression of the embryo was observed after 16–18 h of fertilization. Later, 1–2 high-quality embryos were selected for transplantation 48–72 h after oocyte retrieval according to the patient's status. The routine corpus luteum support was given postoperatively, and the remaining embryos were directly frozen or frozen after blastocyst culture.

#### Outcome measures

1. Baseline clinical characteristics: age, body mass index (BMI), duration of infertility, ovarian volume, antral follicle count, FSH, LH, E2, progesterone (P), anti-Mullerian hormone (AMH), and fasting insulin (FINS).
2. COH: Days of Gn use, total Gn dosage, and trigger time.
3. Sex hormone levels, endometrial thickness, ratios of three endometrial ultrasound patterns on the day of human chorionic gonadotropin (HCG) injection. Endometrial patterns could be divided into three different types [15]: (A) a completely homogeneous, hyperechoic endometrium; (B) an intermediate type with the same ultrasound reflexes as myometrium, and the central echogenic line was not obvious or absent; and (C) a multilayered endometrium consisted of prominent outer and central hyperechoic lines and inner hypoechoic areas.
4. Laboratory results of IVF-ET: cycle cancellation rate, number of retrieved oocytes, fertilization rate, cleavage rate, high-quality embryo rate, and frozen embryo rate.
5. Pregnancy outcomes: clinical pregnancy rate, biochemical pregnancy rate, early abortion rate, rate of multiple births, ectopic pregnancy rate, and number of live births.

### Statistics and analysis

All data were statistically analyzed using SPSS 20.0 software. Enumeration data were expressed as percentage (%), and the χ^2^ test was used for comparison between groups. Measurement data possessing normal distribution were expressed as mean ± standard deviation (SD); independent sample t-test was used for comparison between groups, and non-parametric test was applied when conditions for independent sample t-test were not met. Besides, *p* < 0.05 was considered statistically significant.

## Results

### Clinical baseline characteristics of included patients

The two groups, as shown in Table 1, displayed no significant differences in age, BMI, duration of infertility, ovarian volume, antral follicle count, and baseline levels of LH, FSH, E2, P, AMH, and FINS (*p* > 0.05).

**Table 1:** Baseline characteristics of the patients with polycystic ovary syndrome (PCOS) between the two groups

	Inhibitor group (*n* = 50)	Control group (*n* = 50)	t	*P*
Age (year)	32.02 ± 4.27	31.50 ± 4.71	0.578	0.564
BMI (kg/m^2^)	25.04 ± 2.52	25.42 ± 1.87	-0.851	0.397
Duration of infertility (year)	4.02 ± 1.27	3.94 ± 1.43	0.295	0.768
Ovarian volume (cm^3^)	6.43 ± 1.25	6.57 ± 0.89	-0.646	0.520
Antral follicle count	31.48 ± 4.55	31.52 ± 4.10	-0.046	0.963
FSH (mIU/ml)	6.83 ± 1.08	6.50 ± 0.75	1.727	0.088
LH (mIU/ml)	10.21 ± 1.18	10.74 ± 1.65	-1.836	0.070
E2 (pmol/L)	528.09 ± 120.81	519.54 ± 84.18	0.411	0.682
P (nmol/L)	1.30 ± 0.15	1.25 ± 0.21	1.356	0.178
AMH (ng/ml)	11.72 ± 2.58	11.44 ± 1.56	0.660	0.511
FINS (mU/L)	5.51 ± 1.23	5.42 ± 0.36	0.493	0.624

Measurement data were expressed as mean ± standard deviation (SD)

*FSH* Follicle-stimulating hormone, *LH* Luteinizing hormone, *E2* Estradiol, *P* Progesterone, *AMH* Anti-Mullerian hormone, *FINS* Fasting insulin, *BMI* Body mass index

### Comparison of the mean days of gonadotropin use, mean total gonadotropin dosage, and mean trigger time between the two groups

The mean days of Gn use, mean total Gn dosage, and mean trigger time in the Inhibitor group were significantly lower than those in the Control group (mean Gn days: 9.62 days *vs*. 11.16 days; mean total Gn dosage: 1902.54 IU *vs*. 2373.92 IU; mean trigger time: 3.00 days *vs*. 4.12 days; all *p* < 0.01) (Table 2).

**Table 2:** Comparison of ovarian hyperstimulation between the two groups

	Days of Gn use (d)	Total Gn dosage (IU)	Trigger time (d)
Inhibitor group (*n* = 50)	9.62 ± 1.60	1902.54 ± 267.30	3.00 ± 0.83
Control group (*n* = 50)	11.16 ± 2.40	2373.92 ± 247.22	4.12 ± 1.42
t	-3.771	-9.155	-4.802
P	< 0.001	< 0.001	< 0.001

Measurement data were expressed as mean ± standard deviation (SD)

### Endometrial conditions and sex hormone levels on the day of human chorionic gonadotropin injection between the two groups

There was no significant difference in endometrial thickness and the proportion of endometrial A, B and C morphology on the day of HCG injection between the two groups (*p* > 0.05). However, compared with the Control group, the serum E2 level on the day of HCG injection was significantly lower (*p* < 0.05) while the serum LH and P levels were much higher (*p* < 0.01) in the Inhibitor group (Table 3).

**Table 3:** Comparison of endometrial conditions and sex hormone levels on the day of HCG injection between the two groups

	E2 (pg/ml)	LH (pg/ml)	P (ng/ml)	Endometrial thickness (mm)	Endometrial morphology		
					A	B	C
Inhibitor group	3474.24 ± 300.20	4.33 ± 1.16	1.86 ± 0.31	9.35 ± 1.05	30 (60.0)	14 (28.0)	6 (12.0)
Control group	3641.73 ± 363.44	3.36 ± 0.88	1.55 ± 0.17	9.24 ± 1.10	32 (64.0)	13 (26.0)	5 (10.0)
*t/χ2*	-2.513	4.677	6.228	0.512	0.192		
*P*	0.014	< 0.001	< 0.001	0.610	0.908		

Measurement data were expressed as mean ± standard deviation (SD). Enumeration data are presented as n (%)

*LH* Luteinizing hormone, *E2* Estradiol, *P* Progesterone

### Comparisons of the high-quality embryo rate of patients between the inhibitor and control groups

A total of 69 cycles were performed in the Inhibitor group, including 1 cycle canceled due to suboptimal ovarian response and 68 cycles with successful oocyte retrieval, and the cycle cancellation rate was 1.45%. The average number of oocytes retrieved per cycle was 15.29, and the total number of retrieved oocytes was 1040. Additionally, there were 674 cases (64.81%) of successful fertilization and 614 cases (91.10%) of cleavage. Finally, the Inhibitor group obtained 255 high-quality embryos (37.83%) and 362 frozen embryos (80.09%).

A total of 75 cycles were carried out in the control group, including 2 cycles canceled because of suboptimal ovarian response and 73 cycles with successful oocyte retrieval, and the cycle cancellation rate was 2.67%. The average number of oocytes retrieved per cycle was 15.99, and there were 1167 retrieved oocytes, including 751 (64.35%) cases of successful fertilization and 690 (91.88%) cases of cleavage. Lastly, 221 high-quality embryos (29.04%) and 459 frozen embryos (89.47%) were obtained from the Control group.

Briefly, the Inhibitor group exhibited a much higher high-quality embryo rate and a significantly lower frozen embryo rate than the Control group (*p* < 0.05). However, there were no marked differences in the cycle cancellation rate, number of oocytes retrieved, fertilization rate, and cleavage rate between the two groups (*p* > 0.05) (Table 4).

**Table 4:** Comparison of laboratory results of in vitro fertilization-embryo transfer (IVF-ET) between the two groups

	Inhibitor group (*n* = 50)	Control group (*n* = 50)	*t/χ2*	*P*
Cycle cancellation rate (%)	1.45 (1/69)	2.67 (2/75)	0.261	0.609
Number of retrieved oocytes	15.29 ± 3.25	15.99 ± 2.06	-1.497	0.137
Fertilization rate (%)	64.81 (674/1040)	64.35 (751/1167)	0.050	0.824
Cleavage rate (%)	91.10 (614/674)	91.88 (690/751)	0.278	0.598
High-quality embryo rate (%)	37.83 (255/674)	29.04 (221/751)	11.284	0.001
Frozen embryo rate (%)	80.09 (362/452)	89.47 (459/513)	16.671	< 0.001

Measurement data were expressed as mean ± standard deviation (SD). Enumeration data are presented as n (%)

### Comparison of pregnancy outcomes between the inhibitor group and the control group

The Inhibitor group presented 31 cases (48.44%) of clinical pregnancy, 2 cases (3.13%) of biochemical pregnancy, 2 cases (6.45%) of early abortion, 12 cases (38.71%) of multiple births, 2 cases (6.45%) of ectopic pregnancy, and 15 live births. In the Control group, there were 25 cases (37.97%) of clinical pregnancy, 3 cases (3.61%) of biochemical pregnancy, 2 cases (8.00%) of early abortion, 10 cases (40%) of multiple births, 2 cases (8.00%) of ectopic pregnancy, and 13 live births.

The clinical pregnancy rate of patients in the Inhibitor group was much higher than that in the Control group (*p* < 0.05), while there were no differences in the biochemical pregnancy rate, early abortion rate, multiple birth rate, ectopic pregnancy rate and the number of live births between the two groups (*p* > 0.05) (Table 5).

**Table 5:** Comparison of pregnancy outcomes between the two groups

	Inhibitor group (*n* = 50)	Control group (*n* = 50)	*χ2*	*P*
Clinical pregnancy (%)	48.44 (31/64)	37.97 (25/83)	5.141	**0.023**
Biochemical pregnancy rate (%)	3.13 (2/64)	3.61 (3/83)	0.026	0.871
Early abortion rate (%)	6.45 (2/31)	8.00 (2/25)	0.050	0.823
Multiple birth rate (%)	38.71 (12/31)	40.00 (10/25)	0.10	0.922
Ectopic pregnancy rate (%)	6.45 (2/31)	8.00 (2/25)	0.050	0.823
Number of live births	15	13	0.072	0.788

Enumeration data are presented as n (%)

## Discussion

Most PCOS patients are accompanied by hyperandrogenism and ovulatory dysfunction, such as oligoovulation or persistent anovulation. Therefore, PCOS patients are prone to suffering from infertility compared with non-PCOS patients [16]. The ovaries and adrenal glands secrete excessive hormones in response to hyperandrogenism, resulting in decreased FSH levels, raised LH levels, and an increased LH/FSH ratio [17, 18]. A decline in FSH levels inhibits follicular development, that is, follicles cannot enter the next development stage after developing to a certain extent and eventually fail to mature. Elevation of LH levels is persistent but without periodic fluctuations and LH peak, causing inability to ovulate or abnormal ovulation and then infertility [19]. Weight loss, medical ovulation induction or laparoscopic ovarian drilling have a certain positive effect on ovulation. However, when the above treatment methods fail, IVF-ET is the only possible treatment for PCOS patients willing to get pregnancy. According to statistics, the annual number of IVF-ET treatment cycles performed in China is more than 300,000, ranking first in the world [20]. Hence, the main objective of this study was to investigate the clinical efficacy and application value of TNF inhibitors in PCOS patients undergoing IVF-ET. We found that PCOS patients will face the problems of long COH time, high Gn dosage, low high-quality embryo rate, low clinical pregnancy rate, and high abortion rate during IVF-ET, but TNF-α inhibitors help to improve high-quality embryo rate, clinical pregnancy rate, days of Gn use, total Gn dosage, trigger time, sex hormones on the day of HCG injection. This suggests that TNF-α inhibitors contribute to conception in infertile patients with PCOS undergoing IVF-ET.

TNF-α, as an important inflammatory cytokine, mainly affects insulin sensitivity in peripheral tissues and local androgen levels during the pathogenesis of PCOS [21]. The comparison results in this study proved that TNF-α inhibitor regimen (Inhibitor) was superior to no inhibitor regimen (Control) in reducing the days of Gn use, total dosage of Gn, trigger time, and E2 levels, and increasing serum LH and P levels. It could be conjectured that, through affecting the translocation of glucose transporter-4 and insulin signal transduction, TNF-α inhibitors decreased TNF-α levels in follicular fluid, promoting the process of FSH-induced aromatase activity in granulosa cells, thereby resulting in a decrease in androgen levels of local ovary and the number of follicles [22].

COH is the most critical step in the process of IVF-ET, affecting the number of ovum obtained, ovum quality, number of fertilized ovum, and even the quality of embryos [23]. The quality of embryos is closely related to the clinical outcome of IVF-ET [23]. High basal LH levels are commonly observed in PCOS patients. Generally, high basal LH levels can reduce ovum quality, fertilization rate, and proportion of embryos available through inducing atresia of immature follicles or preovulatory follicular luteinization [24]. Additionally, PCOS patients are a special population mainly manifested in the high number of antral follicles, high basal LH/FSH, and immature follicles despite developing to a certain extent. A great many small follicles are recruited during COH, and a large number of mature ovums can be obtained after ovulation induction. However, a high level of estrogen in the ovary can easily trigger ovarian hyperstimulation syndrome, especially after pregnancy [25, 26]. The high level of estrogen internal environment in the ovaries of PCOS patients can greatly increase their ovarian sensitivity to Gn and even can be response to endogenous Gn, resulting in recruitment of a large number of follicles [27]. In short, PCOS patients treated with IVF-ET are prone to uncontrolled ovarian response during COH. These particularities cause PCOS patients to present a high number of oocytes retrieved, low fertilization rate, and high risk of ovarian hyperstimulation syndrome during IVF-ET treatment, bringing difficulties to IVF-ET for PCOS patients. In this study, compared with the Control group, the Inhibitor group presented significantly increased high-quality embryo rate, and clinical pregnancy rate, but the two groups presented no significant differences in the cycle cancellation rate, number of oocytes retrieved, fertilization rate, cleavage rate, biochemical pregnancy rate, early abortion rate, multiple birth rate, ectopic pregnancy rate and number of live births. Shortly speaking, various factors affect embryo quality and pregnancy outcomes, rather than just the ovulation induction regimen used during COH.

There are some limitations in this study. For instance, this paper is a retrospective study with a limited sample size. Therefore, multicenter, large-sample prospective studies are needed to further improve the investigation.

## Conclusion

For PCOS patients undergoing IVF-ET, the TNF-α inhibitor regimen contributes to improving days of Gn use, total Gn dosage, trigger time, sex hormone levels on the day of HCG injection, and clinical pregnancy rate. In other words, TNF-α inhibitors have a certain application value for IVF-ET in infertile women with PCOS.

## Data Availability

The dataset generated and/or analysed during the study are available from the corresponding author on reasonable request.

## Abbreviations

TNF: Tumor necrosis factor
IVF-ET: In vitro fertilization-embryo transfer
PCOS: Polycystic ovary syndrome
Gn: Gonadotropin
HCG: Human chorionic gonadotropin
COH: Controlled ovarian hyperstimulation
GnRH: Gn releasing hormone
FSH: Follicle-stimulating hormone
LH: Luteinizing hormone
E2: Estradiol
P: Progesterone
AMH: Anti-Mullerian hormone
FINS: Fasting insulin
